# Supplementary material for: Brain vasculature accumulates tau and is spatially related to tau tangle pathology in Alzheimer’s disease
Source: Acta Neuropathol. 2024 Jun 17;147(1):101. doi: 10.1007/s00401-024-02751-9 (PMC11182845; doi:10.1007/s00401-024-02751-9)
Supplement: Supplementary file 2 — Supplementary file2 (DOCX 382 KB) [file 401_2024_2751_MOESM2_ESM.docx]

**Supplementary Information**

**Title:** Brain Vasculature Accumulates Tau and Is Spatially Related to Tau Tangle Pathology in Alzheimer’s Disease

**Authors:** Zachary Hoglund^1^, Nancy Ruiz-Uribe^1,2^, Eric del Sastre^1^, Benjamin Woost^1^, Elizabeth Bader^1^, Joshua Bailey^1^, Bradley T. Hyman ^1,2^, Theodore Zwang^1,2,ꝉ,^*, Rachel E. Bennett^1,2,ꝉ,*^

**Affiliations:**

^1^ Department of Neurology, Massachusetts General Hospital, Charlestown, MA, USA

^2^ Harvard Medical School, Boston, MA, USA

^ꝉ^ These authors contributed equally to this work.

* Address correspondence to these authors at the Department of Neurology, Massachusetts General Hospital, 114 16th Street, Charlestown, Massachusetts, USA; Tel: 617-726-1263; E-mails: [rebennett@mgh.harvard.edu](mailto:rebennett@mgh.harvard.edu), tzwang@mgh.harvard.edu

**Supplementary Methods:**

**SDS-PAGE Western Blotting.** For standard SDS-PAGE, 10 µg protein per sample was mixed with 10x NuPAGE sample reducing agent (Invitrogen, cat no. NP0004) , 4x NuPAGE LDS sample buffer (Invitrogen, cat no. NP0007), and boiled for 5 minutes at 95°C. The resulting samples, and a SeeBlue Plus2 pre-stained protein standard (Invitrogen, cat no. LC5925) was loaded on a 4-12% Bis-Tris gel (Invitrogen, cat no. NP0323BOX) in MOPS SDS running buffer (Invitrogen, cat no. NP0001) and run at 120V for 1.5 hours. Proteins were then transferred to nitrocellulose at 90V for 1.5 hours. Membranes were briefly dried and then rehydrated in TBS, blocked with Intercept TBS blocking buffer (Licor, cat no. 927-60001) and incubated for 3 hours at room temperature with anti-pT217 in Intercept T20 antibody diluent (Licor, cat no. 927-65001), followed by a 1-hour incubation with goat anti-rabbit 800 (Licor, cat no. 926-32211) and visualized on a Licor imaging station. The blot was then stripped and re-probed with anti-Glut1 and anti-rabbit 800 following the same method. A second blot was incubated in Revert 700 total protein stain (Licor, cat no. 926-11011) according to the manufacturer’s instructions.

**Supplementary Data:**


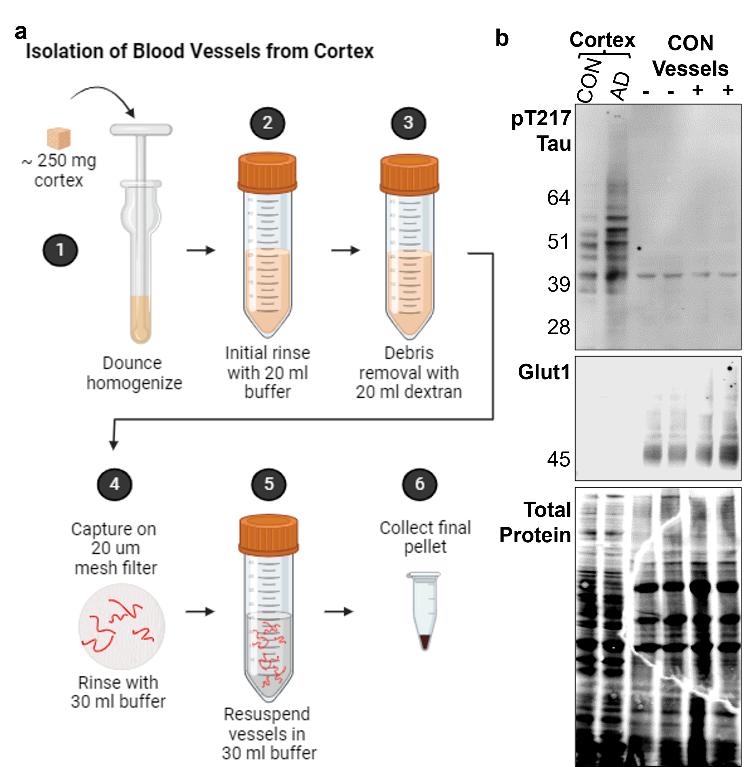


**Supplementary Figure 1: Isolation of blood vessels from cortex does not increase tau association with the vascular compartment.** (**a**) An overview of the method used to isolate blood vessels from cortex. (**b**) In a control experiment, cortical homogenates were prepared from a control or AD brain. Blood vessels were isolated in four technical replicates from the control cortex. After isolation, two of these replicates were incubated with 25 µg of AD cortical lysate for 30 minutes (the approximate time required to complete steps 1-3) and then vessels were recaptured on mesh and steps 4-6 were repeated to yield a final vessel pellet that had been incubated with or without AD brain lysate. This indicates that blood vessel isolation does not artifactually result in enhanced vascular tau. GLUT1 labeling confirms enrichment for blood vessels versus cortical homogenates. Total protein is also included as a control.

**Supplementary Movie 1: Closeup view of CAA-positive vessels from donor AD 6.** Video shows three dimensional images of GLUT1 labeled blood vessels (magenta), tau (white), and amyloid beta (yellow) from dashed box in Figure 3A. Tau is present within dystrophic neurites surrounding amyloid beta plaques but not near amyloid beta-positive leptomeningeal blood vessels that are visible on the right-hand side of the image.
